# Access and efficacy of university mental health services during the COVID-19 pandemic

**DOI:** 10.3389/fpubh.2023.1269010

**Published:** 2023-12-20

**Authors:** Beverly Wagner, Yaser Snoubar, Yousif S. Mahdi

**Affiliations:** ^1^Social Work Department, Texas Woman's University, Denton, TX, United States; ^2^Social Sciences Department, Qatar University, Doha, Qatar

**Keywords:** COVID-19 pandemic, mental health access, university students, telehealth, higher education support services

## Abstract

**Objective:**

This study sought to understand the mental health issues, mental health support and efficacy of that support among university students.

**Participants:**

All students enrolled in a College of Arts and Sciences at one mid-size university received an email that contained a link to an anonymous, online questionnaire developed and disseminated through PsychData. 162 students completed the questionnaire.

**Methods:**

Mixed methods: Data was summarized using descriptive analysis, testing for significance, testing for differences, and content analysis.

**Results:**

Participants reported high levels of anxiety (76%) and depression (65%). Results indicated that participant demographics were associated with types of mental access, and support. Unexpected results included lack of knowledge or information on cost, and how to access mental health services hindered access for participants, and although telehealth was the most widely used support, in contrast to other studies, participants indicated a preference for face-to-face mental health services.

**Conclusion:**

Results highlight the need for improving communication about and access to mental health services in higher education Recommendations and implications for policy and support services are provided.

## Introduction

College students have shown an increased risk of psychological issues across a broad range of mental health problems that predate the pandemic (1). However, mental health concerns increased significantly across a diverse range of global, geographic regions and college student populations during the pandemic leading to speculations that college students are “uniquely vulnerable to mental disorders and stress” (p. 457) particularly during public health crises (1). Data from Texas A&M University, for example, found that out of 2031 undergraduate and graduate students, 48.14% showed moderate-to-severe levels of depression, 38.48% with moderate-to-severe levels of anxiety, and 18.04% exhibited suicidal thoughts during the pandemic (2). Lee et al. (3) noted similar results in their study of 200 college students, in which 60.8% of respondents reported an increase in anxiety and 54.1% reported an increase of depression since the onset of the pandemic. Furthermore, in a smaller investigation of predominately female college students, results revealed that students who completed measures of mental health symptoms and stress during the pandemic, reported more symptoms of depression, stress, and alcohol use than a sample of students who completed the same measures before the pandemic (4). Other studies examined the effects of the pandemic among specific college disciplines such as medical students who already were experiencing higher rates of mental issues before the pandemic. The pandemic exacerbated overall rates of depression (18.6%) and anxiety (47.8%), and higher rates associated with being a female medical student, and in the first term of study (5). Research among engineering students highlighted higher levels of distress among historically underrepresented engineering students indicating potential intersectional factors that impacted mental health (6). Additionally, research that investigated the impact of COVID-19 stay-at-home orders on student mental and behavioral health outcomes found that scores on anxiety and depression scales were statistically higher than they had been prior to the pandemic (7).

Despite this uptick in mental health concerns, and pandemic-related research suggesting that the delivery of mental health support had changed to mitigate disparities in mental health-care provisions, use of mental health services among college students remains low (8–10). Moreover, it is unclear what the COVID 19 related mental health service-use outcomes are for college students and whether the shift in campus and community treatment processes and policies improved service access and efficacy. This research investigated the mental health needs of a diverse range of students attending a midsize university located in the Southwestern region of the United States (US), the mental health support received, and the efficacy of that support during the COVID-19 lockdown and continuing COVID waves. In this article, associations between mental health issues, demographic characteristics, access to and efficacy of mental health services are examined, and implications and recommendations for college and university mental health services and broader institutional responses are provided.

## Literature review

In the spring of 2020, the World Health Organization, (WHO) declared COVID 19 a pandemic that prompted lockdowns worldwide (11). By April of 2020, higher education institutions in 185 countries were closed, online learning ubiquitously replaced face-to-face teaching, and 1,100 U.S. colleges and universities within all 50 states canceled face-to-face classes (11, 12). Student mental health was impacted globally by a range of issues related to these sudden changes such as the rapid transition to online learning, and social isolation (13). Other risk factors included students’ anxiety about their academic futures, the economic climate, where they lived, and decisions to move from on-campus housing to other housing (14). For example, higher levels of anxiety and depression were noted among Italian students when compared to general workers within the larger population particularly on such variables as one’s own health concerns and fear of COVID which the researchers hypothesized could be linked to anxiety about their futures (15).

Due to the lock down, students were isolated and had to adjust to a lifestyle that diminished real world interactions, and increased internet usage (16, 17). Recent research highlights correlations between social confinement and traumatic distress. For example, 21.4% of students who sought help at a university counseling center experienced the lockdown as traumatic. Risk factors included an “all or nothing thinking style” and the length of time spent in the lockdown (18). Addictive behaviors, such as internet addictions, were also exacerbated by more time spent on computers and electronic devices due to distance learning and to cope with anxiety (19). Notably, the prevalence of depression observed among students was also linked to distance learning (14, 17). As the mode of education changed, students were more likely to experience higher levels of anxiety, depression, substance abuse, and eating disorders particularly as the confinement lengthened (20, 21). For instance, distance learning increased Indonesian students’ feelings of loneliness which negatively impacted their mental health (17).

Studies from the Southern region of the US noted similar and additional results. For example, isolation due to the quarantine, appeared to exacerbate preexisting mental health disorders such as PTSD or depression (4). Substance misuse such as tobacco, alcohol, and marijuana were also linked to mental illnesses including depression, anxiety, hopelessness, and suicidality (4, 19, 22). For college students who were already drinking before the pandemic, obstacles created by online learning were associated with more significant stress and increased alcohol usage (23, 24). These substance-related health risks were public health concerns, indicating a need for a more thorough understanding of the underlying causes, including how people coped with COVID-19-related stress. Indeed, a better awareness of student risk and protective factors during the pandemic could have influenced university support services to increase outreach services and encourage proactive student behaviors, such as seeking social support and mental health services (25).

Similar to the wide range of health care systems worldwide, mental health care and related policies rapidly adapted to meet the needs generated by the COVID-19 pandemic. This shift in mental health services and policies that recommended, and often mandated telehealth presented both innovative opportunities for service users to utilize technology to generate mental health support as well as risks (26). These Telehealth innovations and risks impacted college and university students both positively and negatively. Positive impacts included alternative modes of accessibility with the potential to reach a wider group of students. Yet telehealth was not consistently available and negatively affected those who struggled with internet accessibility issues (26–28). Moreover, issues of digital access disproportionately impacted US students of color such as African American, Latinx, Indigenous, and Multiracial students, during the pandemic who returned to communities unduly impacted by digital access issues (29). Michaels et al. (28) add issues of confidentiality and finding private spaces to access telehealth appointments as problems for students. Nevertheless, the authors reported that students who accessed treatment at an outpatient mental health clinic indicated an overwhelming preference for telehealth due in part to the convenience of this mode of service.

Other issues also interfered with accessing mental services at colleges and universities. Although, telehealth emerged as a way for colleges and universities to provide mental health and victim services to students, some US campus resources were no longer available to students due to funding deficits (30). This was problematic as particularly the decade before the pandemic, the mental health of students in US higher education was a growing issue as evidenced by the 2018 US National College Health Assessment which documented that 62.3% of respondents reported overwhelming anxiety and 41.4 reported beings so depressed they could not function anytime within the previous 12 months (31).The pandemic exacerbated these risks and increased the vulnerability of students to the psychological impact of COVID-19. However, studies indicate even if services are increased, student education and outreach about what services are available, and what mental health and mental health treatment entails, is needed to increase accessibility (32, 33). This is particularly important as mental health issues are a primary obstacle to academic success (9). Nevertheless, only a small percentage of young adults seek professional care and/or care from college and university counseling centers (10, 34). Access and outreach of university mental health services is not only an ethical consideration but also a legal one. Tanabe et al. (33) emphasize that case law recently determined that US universities have duty care which includes protecting students from foreseeable harm. Thus, continued advocacy for fully funded and policy-supported higher education institutional outreach and mental health services in various formats remains a priority (20, 35).

Student identities also played a role in the prevalence of mental health concerns and ability to access care. Within the Southern region of the US, Correia et al. (36) found that restricted access to health care, COVID-19 risk, and disparities in healthcare access, wages and housing, negatively impacted communities, and students of color. Differences in health beliefs and the perceived threat of COVID-19 may have also affected prevention and treatment support (32). Hersch et al. (37) adds the issue of digital disparities among US college students, which unevenly impacted students of color. The intersectionality of identities such as mental illness, racial/ethnic minority, and gender identity, increased the risk of discrimination, health disparities, and heighten health risks (3, 38). Indeed, perceptions of stigmatization due to mental illness and previous discrimination experiences were noted as significant barriers to utilizing mental health services (10, 39). Students who were underrepresented in academia, from underrepresented ethnic groups, new to college, and not residing on campus had a particular need for assistance (40). For students of color, race-based stressors exacerbated by the COVID 19 pandemic remain a particular concern. This could include race-related discrimination, such as discrimination experienced by Asian American students calling for mental health approaches that account for diverse experiences (32).

Thus, the objectives of this study were to understand the responsiveness of student mental health services during the pandemic, the mental health supports students were more likely to access, the efficacy of that support, and gaps or barriers to mental health services. Research questions included:

i) What mental health issues, if any, had participants experienced?
ii) If mental health issues were experienced, what support did participants receive?
iii) What was the efficacy of the support received?
iv) If support was not received, what were the reasons?

## Materials and methods

### Participants and setting

Data was collected from undergraduate and graduate students enrolled in a College of Arts and Sciences program or course (*N* = 162) at a midsize, public university located in the Southwest Region of the United States during January–April 2022. The university is located in a suburban setting within a larger metropolitan area. The ethnic diversity of the approximately 16,000 student population is similar to other US metropolitan and Southwest regional universities, and ethnically diverse students make up over 55% of the population. The university offers a wide range of majors but is known for its programs in nursing, education, health care, and arts and sciences (41).

A full population questionnaire was distributed to all students enrolled in the College of Arts and Sciences. This college was selected to recruit participants due to the college’s historical, high student enrolment. Inclusion criteria included enrolment as an undergraduate or graduate student in the College of Arts and Science, began attending college on or after Fall 2020, 18 years old or above. Exclusion criteria included a lack of questionnaire completion.

### Ethics statement

The study received approval from the university’s institutional review board (IRB), and all participants received a written study description and informed consent information prior to completing an anonymized survey. The study was initiated by a collaboration of social sciences faculty with interests in student mental health. Faculty were from the institution, and a partnering international university.

### Research design and data collection

Using an online questionnaire design, data was drawn from a sample of self-reporting college participants. The questionnaire was designed by the researchers in Psych Data and distributed through a link imbedded in emails or email flyers sent directly to all undergraduate and graduate students enrolled in a College of Arts and Science program or course.

An embedded mixed method design was used, a qualitative component embedded in a quantitative questionnaire. This questionnaire included a combination of dichotomous, Likert, multiple answer, forced choice, fill in the blank, and open questions. Preliminary questions gathered demographic data as well as assessed if participants had been formally diagnosed with a mental health condition since the advent of the COVID 19 pandemic. Open questions explored participant suggestions for improving and/or developing access and efficacy for services based upon their experiences. Additional surveys to assess students’ present mental states were not included in the study design as the intent of the questionnaire was to understand mental health issues experienced by students, the support students received, and the efficacy of that support if they experienced mental health issues. Examples of questions can be found in Table 1.

**Table 1:** Question examples.

Assessment of mental health issues	Assessment of mental health support	Efficacy of that support
Mental disorder diagnosis prior to the pandemic	Mental health support or treatment received in the last 18 months	Reasons for not using support at the time one experienced a mental health issue or condition.
Mental disorder diagnosis during the pandemic	Where support was received	If treatment was received, level of satisfaction
Mental conditions one has experienced during the last 18 months	Mode of support such as face-to-face, telehealth, etc. Frequency of Support	Type of preferred mental health mode of support

### Data analysis

The variables assessed in the online questionnaire measured participants mental health past and recent history during the COVID-19 pandemic as well as the type and form of treatment provided, treatment follow-up, gaps and/or disruptions to treatment, and treatment efficacy and/or preferences. Descriptive analysis and Kruskal-Wallis *H* test for comparing the average frequency of support or treatment received across different ethnic groups was utilized. A factor analysis assessed the internal consistency of the questionnaire. Based on 59-items (excluding qualitative responses and items that had zero variance), the Cronbach’s alpha score was α = 0.772. All analyses were performed with SPSS version 28.

Content analysis was used to analyze data obtained from the questionnaires’ open question exploring participant suggestions for improving access and efficacy of services based upon their experiences. We independently coded the data using a deductive, top-down approach coding and categorizing responses based upon frequency of repeated themes within the data that aligned with the research questions addressing access and efficacy of services (42). Responses to the open questions were then linked to the frequencies and percentages derived from the quantitative data or as Braun and Clark (42) contend, content analysis allows for frequency counts. To establish trustworthiness and validity of the qualitative data, data were verified through a triangulation of quantitative and qualitative data. An audit trail was created that included the data files of the questionnaires, and interrater agreement between the researchers.

## Results

### Research question i: what mental health issues, if any, had participants experienced?

**Figure 1:**
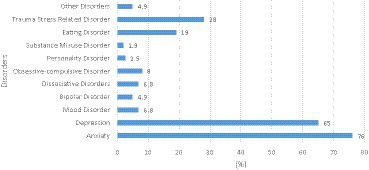
Prevalence of mental health disorders or conditions among students (*N* = 162).

The prevalence of various mental health issues self-reported by students indicated high rates of anxiety and depression. Anxiety was the most reported disorder, affecting 76% of participants. Depression was the second most common, with a prevalence of 65%. Trauma Stress Related Disorder was reported by 28% of the students, and eating disorders affected 19% of the sample. Personality Disorder and Substance Misuse Disorder were the least common disorders in the sample, with prevalence rates of 2.5 and 1.9%, respectively (Table 2; Figure 1).

**Table 2:** Prevalence of mental health disorders or conditions among students (*N* = 162).

Disorders	*N*	%
Anxiety	123	76
Depression	105	65
Mood disorder	11	6.8
Bipolar disorder	8	4.9
Dissociative disorders	11	6.8
Obsessive-compulsive disorder	13	8
Personality disorder	4	2.5
Substance misuse disorder	3	1.9
Eating disorder	30	19
Trauma stress related disorder	46	28
Other disorders	8	4.9

### Research question ii and iii: if mental health issues were experienced, what support did participants receive? Efficacy of that support?

In a sample of 162 female students at a U.S. women’s university, the majority of participants, 68%, reported receiving no support for their mental health concerns. Among those who did receive support, 8.7% accessed resources through the university counseling center or related university resources, while 23.3% sought help from community mental health providers.

In terms of the types of support received, face-to-face counseling was utilized by 9.9% of the students, telehealth/online counseling by 16%, and medication management by 13%. At-home visits and group therapy in person were less commonly reported, with utilization rates of 1.2 and 2.5%, respectively. Participants who received support and treatment also rated their satisfaction with treatment and its influence on their coping with mental health issues. Fifty percent (*N* = 50%) indicated satisfaction with treatment, while (*N* = 50%) indicated neutral, dissatisfied or very dissatisfied (Table 3).

**Table 3:** Mental health support location (*N* = 103).

Support	*N* (%)
Mental health support location	
University counseling center or related university resources	9 (8.7)
Community mental health providers	24 (23.3)
No Support	70 (68)
Type of support	
Face-to-face counseling	16 (48.5)
Telehealth/Online counseling	26 (78.8)
Medication management	21 (63.3)
At-home visits	2 (6.1)
Group therapy in person	4 (12.1)

### Research question iv: if support was not received, what were the reasons?

**Figure 2:**
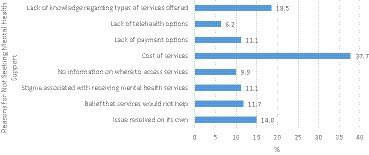
Reasons for not accessing services (*N* = 162).

For students who did not receive support for mental distress within the last 18 months, various reasons were reported for not utilizing mental health services. The most common reason cited was the cost of services (37.7%). Lack of knowledge regarding types of services offered was reported by 18.5% of the students, followed by lack of payment options 11.1% and stigma seeking services which was also cited as a reason by 11.1% of respondents (Table 4; Figure 2).

**Table 4:** Reasons for not accessing services (*N* = 162).

Reasons for not accessing services	*N*	%
Issues resolved on its own	24	14.8
Belief that service would not Help	19	11.7
Stigma associated with receiving mental health services	18	11.1
No information on where to access services	16	9.9
Cost of services	61	37.7
Lack of telehealth options	10	6.2
Lack of payment options	18	11.1
Lack of information on the type of services	30	18.5

### How does the average frequency of support or treatment received vary by ethnicity?

The Kruskal-Wallis test results revealed significant differences in the average frequency of support or treatment received among different ethnic groups (H (4) = 12.707, *p* = 0.013). The median frequency of support or treatment received for the Hispanic/Latinx group (Mdn = 3) is higher than the median for other ethnic groups (Mdn = 2 for Caucasian/White, African American, Asian, and Other). This indicates that, on average, Hispanic/Latinx students received mental health support or treatment more frequently than students from other ethnic groups (Table 5).

**Table 5:** Kruskal-Wallis test for differences in average frequency of support or treatment by ethnicity.

Ethnicity	*N*	Mean Rank	Median	Kruskal-Wallis H	df	Value of *p*
Caucasian/White	84	75.71	2	12.707	4	0.013
Hispanic/Latinx	35	104.33	3			
African American	18	76.36	2			
Asian	16	79.13	2			
Other	9	61.22	2			

### Qualitative open responses

The following themes reflect a content analysis of student open responses to questions regarding suggestions for improving access and efficacy of services. Themes included a lack of Information about services, the cost of services, and a lack of options. Students expanded on quantitative responses that addressed reasons for not accessing services and/or efficacy of the services as well as suggested improvements. The frequency of their responses generated themes that aligned with research question iii: efficacy of support received and expanded on research question iv. reasons for not accessing services.”.

#### Lack of information

Similar to quantitative responses in which 28.4% of students indicated that they did not pursue mental health services due to lack of knowledge on types of mental health services or where to access services, open responses elaborated on these concerns. Student responses discussed not knowing of health or mental health services offered on campus. As one participant stated, “*I also did not know we had service*s.” A lack of education on service options was also described by another participant: “*In truth when I was struggling the most I needed information on my options*.” Other students talked about the lack of information on the campus regarding mode of services offered, “*clear communication about the mode of services provided would be helpful*!” payment options if students were referred off campus, and education in general regarding mental health treatment. Finally, how to get started with treatment was a common theme among participants.

#### Cost of services

While almost 48.8% of the participants who were not receiving treatment indicated cost of services or lack of payment options as major deterrents, open responses provided a more nuanced understanding of cost issues. Multiple responses indicated that participants struggled when seeking services at the university counseling center due to lengthy wait times, particularly during COVID pandemic. One participant described struggling with depression and suicidal thoughts and being placed on a wait list that lasted months. Another added, “*University Counseling Center resources are great, but many students are unable to take advantage of it due to the limited number of sessions available for scheduling and amount provided to students each semester*.” Still others described being referred to community provides due to complex mental health issues and being placed on wait lists again due to a lack of mental health providers in the area.

For participants who were referred to community providers or participants who could not wait for university counseling services, cost/payment options became an issue. As one participant stated, “*paying for services wasn’t feasible due to not currently working while in school*.” Another added, “*More providers are definitely needed at rates that students can afford*.” Lack of insurance or insurance restrictions were other limitations or as one participant emphasized “*making quality mental health services more accessible to people that cannot afford*.” Finally, the lack of financial ability to pay insurance co-payments was another common issue.

### Lack of options and accessibility

An emergent finding from the qualitative data was a perceived lack of options to access mental health services. Stemming in part from lengthy wait times at the University Counseling Center, cost when seeking services off campus, and a lack of payment options, participants perceived no significant options for mental health treatment. As one respondent stated, “*Many people I know gave up on getting mental health services on campus because of the red tape and the waiting times*.” Other participants who were successful in accessing appointments with the university counseling center described a lack of follow through. “*I sought services through the school and they told me that my problems were too serious for the services that they offer. They referred me to another counselor outside of the school and she told me the same thing, so I gave up seeking treatment*,” When turning to resources off campus, cost immediately became a deterrent: “*The only thing that hinders my ability to seek professional help is money and how expensive it is*.”

#### Telehealth

Although 6.2% of students indicated they were unable to telehealth options, 78.8% of those who were able to access services reported using telehealth. Telehealth services were described as helpful by some respondents and undesirable by others. Several students indicated being pleased with the university counseling center services, and telehealth sessions. Students also indicated that telehealth visits with community providers were helpful and one described being able to attend therapy for the first time due to the flexibility of telehealth. Yet others indicated a lack of interest in telehealth. As one student described “*Last time I tried to use the university counseling center it was online only and did not help me facilitate a therapeutic connection in the way I needed*.” Others talked about their difficulties in finding private spaces for telehealth.

## Discussion

Anxiety and depression are among the psychological issues that grew in prevalence during the COVID-19 pandemic. The present study depicted similar experiences among students at Southwestern University. Yet this study also found that the efficacy of mental health services was impacted significantly by participants lack of knowledge about services or information on where to access services, wait times at the university counseling center, and cost of off campus mental health services.

Similar to earlier studies, the majority of participants in the present study did not have a clinical diagnosis, yet approximately 76% reported experiencing anxiety and 65% reported experiencing depression (3, 38). Regionally, these rates are comparable to other universities in the Southwest, such as one study of 195 participants signifying increased anxiety and stress (71%) (9). The present study data is also comparable to National trends. The 2022 Center for Collegiate Mental Health’s (CCMH) annual report, which collected data from 684 college and university counseling centers, reported 68.8% of students receiving services indicated COVID 19 negatively impacted their mental health (43). Notably, annual increases were identified in the areas of trauma, social anxiety, and although anxiety remained unchanged from the previous year, it continued to be the most common problem experienced by students. Conversely, the 2022–23 US Healthy Minds study indicated higher levels of depression than anxiety with 41% of 76,406 respondents reporting depression symptoms and 36% reporting symptoms of anxiety (44). The present study echoes these national trends in reporting higher rates of anxiety and depression. These mental health issues could be due the mode of study during the pandemic and the sudden switch to exclusively online teaching methods, and concomitant stressful workloads (45, 46). Additionally, student anxiety has been significantly correlated with anxiety about the future and fear of contracting COVID-19 (45, 47).

Supporting earlier studies, the present study indicated that Telehealth was the most popular type of mental health care during the pandemic (28). Hersch et al. (37) suggest this could be due to ease of access offered by telehealth which increases both attendance and participation. However, participants in the present study expressed a desire for in-person counseling, or at least some in person counseling, as one participant expressed. “*it was only* via *tele-health and at the time I wanted My first appointment to be face to face.”* This contrasts with some studies where levels of satisfaction with telehealth were comparable to face-to-face counseling (27, 28). Telehealth services could also present other disadvantages for those who need it most. Digital disparities such as access to necessary technology (and knowledge of how to use it), internet access, and finding private spaces to access counseling are issues noted in other research studies (8, 37). Nevertheless, the use of technology and telehealth is also a possible response to college students’ mistrust, stigmatization, and reluctance toward utilizing mental health treatment (37, 48).

Like many college and university campuses, the university and university counseling center highlighted in this study, delivered services primarily through HIPPA compliant, virtual platforms during the pandemic through the present and emphasized delivery of culturally responsive mental health services. Yet, a significant portion of students in this study did not receive support for their mental health issues, and less than 10% utilized the university counseling center. Comparable results are found in various other studies reporting significant increases in student anxiety and depression during the pandemic, but low usage rates of mental health services, on or off campus (3, 10, 44, 49). However, 28.3% of participants in the present study indicated that it was a lack of information on types of services or where to access services that negatively influenced their ability to seek support. As one participant emphasized “*it would help a lot to advertise these services.*” Mohlmann and Basch (50) argue the importance of clear university messaging, and ways that consistent messaging can build understanding of support and build resilience during crises such as the COVID-19 Pandemic. Consistent and clear messaging describing the types of counseling support services offered on campuses, cost or no cost of services, and mode of services offered such as telehealth and face-to-face could positively influence student access of services. Yet participants who were aware of the university support services reported additional issues of wait times, and if referred off campus, were again impacted by wait times as well as cost of the services. Lee et al. (49) adds issues of out of state and international students who would not be able to use virtual counseling due to out of state restrictions for licensed counselors as well as remote access problems. However, nationally, issues of university counseling wait times were steadily increasing even before the pandemic due to the rise in mental health problems among students and lack of providers (37). In light of the pandemic related negative effects on students’ mental health, funding that provides for greater access to mental health care, such as delivery of mental health services in multiple formats to ensure students’ well-being and safety, should be prioritized as much as, if not more than, their education (20, 35).

This research also revealed which participants were using on or off campus mental health services and which participants were not. In the present study, it was Hispanic/Latinx students who received the most support. Possible explanations include what Kessler et al. (51) (p. 15) refer to as “the increased risk of pandemic related stressors” and post COVID disasters for disadvantaged groups. For students of color this included carrying an unequal burden of financial stress, limited healthcare resources, illness and death impacting them, their families, and their communities during the pandemic (3, 38). Other research supports findings that students with the most psychological distress do seek support from university counseling centers with few differences in utilization among racial and ethnic groups (10, 49). For example, similar results to the present study were reported by Lee et al. (49) who found usage of on campus mental health services was 3.9% higher for African American students and 2.0% higher for Hispanic students than white students. Contrasting studies, however, have indicated that students of color are historically less likely to access mental health services due to negative views of mental illness and/or treatment and stigma beliefs related to mental illness (52, 53). Indeed, 11% of participants in the present study indicated that stigma was a reason for not seeking services. Other potential explanations for why Hispanic/Latinx students were more likely to seek and obtain services include that the institution where the study took place was a Hispanic Serving institution, so although students overall indicated a lack of knowledge of types of services, some messaging targeting this group was successful.

### Implications for policy and services

Although the COVID-19 pandemic transformed the delivery of mental health services internationally, gaps remain in higher education. The following service and policy implications and recommendations address accessibility gaps, improving outreach, and reducing the stigma of seeking services. These recommendations also address United Nations sustainability development goals (SDG) 3 and 4: Good Health and Well Being, and Quality Education (54).

Results from this study indicated that a lack of knowledge regarding the services was a major deterrent. When addressing mental health on college campuses, the American Council on Education called for university leaders to also focus on consistent outreach and communication with students regarding mental health, wellbeing, and services available to them (55). At the level of on campus services, as on-campus counseling is often free to students, university support services can ensure that students are aware of the services that do exist. An additional awareness strategy could be developed such as a mobile app that includes a map of mental health providers on and off campus, service descriptions, and online appointment scheduling (56).

Yet more than a lack of knowledge, wait times can significantly impact participants ability to access services and in turn impact the efficacy of campus counseling services to meet existing needs. Lack of funding, staff shortages, and increasing numbers of students seeking services all impact mental health support and delivery. Thus, offering services in different formats, that include telehealth and the infrastructure to support it, could provide greater access and flexibility for both counselors and students, and potentially ease wait times (37, 48). Other changes could include easing state restrictions for counselors delivering services to students/clients that are out of state. Examples include some potential easing of restrictions that allow university-based counseling centers to provide remote services beyond state borders, and interstate compacts that allow counselors to deliver services in other states that recognize the compact (37).

Finally, to reduce stigma and boost outreach efforts and care usage, higher education institutions can provide culturally relevant mental health education for students (53). This should include awareness programs that address stigma surrounding mental illness and challenge myths about mental illness, and seeking treatment (52). Subsequently, understanding social and cultural diversity issues and how these issues affect seeking mental health services is also a crucial part of delivering mental health services to student populations.

### Implications for future research

Future studies should examine mental health access among college campuses to strengthen policies and services that support student mental health. A comparative study among colleges and universities in various regions or nationally, could elucidate issues and best practices to address access of mental health services. Additional research is also needed to understand student mental health access and outcomes for students who are referred off campus. Finally, telehealth was the mode of support that study participants said they relied on the most, yet it was also viewed as a deterrent by some. Longitudinal research that compared formats of mental health delivery, usage rates and mental health outcomes among different racial and ethnic groups within university support services could strengthen both access and efficacy of services.

### Limitations

The questionnaire distribution was limited to one Southwestern university, and the college of arts and sciences. Although this study cannot be generalized to other university contexts, the study findings were similar to earlier studies that found a high prevalence of anxiety among participants and higher usage rates among specific groups (3, 49). This study also had a risk of sampling bias as the sample was a convenience sample, and participants with an interest in mental health, mental health history or were in distress, may have been more likely to complete the questionnaire. Additionally, mental health status or ability to access services could have changed for participants over time. Thus, a longitudinal study design would have the potential to capture data regarding efficacy and access of mental health services over time.

## Conclusion

Despite high rates of anxiety, depression, and stress among college students during the COVID 19 pandemic, student access to services was hampered by a lack of understanding or knowledge about services, and/or on campus waiting lists or off campus service costs. This study can inform university mental health policy and services to increase outreach and access of mental health services. This study also addresses the 2030 UN Agenda for Sustainable Development by elucidating obstacles to student wellbeing (SDG3) which could negatively impact students’ ability to achieve a quality education (SDG4) (54). Offering mental health services in different formats to increase flexibility as well as fully funding college and university counseling services could provide greater access and address stigma that may prevent some students from seeking services. Yet, some participants who could access telehealth services still preferred face-to-face counseling, suggesting unexplored obstacles in the provision of mental health services for college students, a need for further research on the types of mental health delivery that best meet the needs of diverse student populations, and a modification in university policies to address service access and delivery.

## Data availability statement

The raw data supporting the conclusions of this article will be made available by the authors, without undue reservation.

## Ethics statement

Ethical review and approval was not required for the current study in accordance with the Texas Woman’s University’s Institutional Review Board. Written informed consent for participation was not required for this study in accordance with the national legislation and the institutional requirements.

## Author contributions

BW: Conceptualization, Data curation, Formal analysis, Funding acquisition, Investigation, Methodology, Project administration, Resources, Software, Supervision, Validation, Visualization, Writing – original draft, Writing – review & editing. YS: Conceptualization, Data curation, Formal analysis, Funding acquisition, Investigation, Methodology, Project administration, Resources, Software, Supervision, Validation, Visualization, Writing – original draft, Writing – review & editing. YM: Conceptualization, Data curation, Formal analysis, Funding acquisition, Investigation, Methodology, Project administration, Resources, Software, Supervision, Validation, Visualization, Writing – original draft, Writing – review & editing.
